# Living with clipped wings—Patients’ experience of losing a leg

**DOI:** 10.3402/qhw.v8i0.21891

**Published:** 2013-10-14

**Authors:** Annelise Norlyk, Bente Martinsen, Klaus Kjaer-Petersen

**Affiliations:** 1 Faculty of Health Sciences, Department of Nursing Science, School of Public Health, Aarhus University, Aarhus, Denmark; 2 Department of Orthopedic Surgery, Aarhus University Hospital, Aarhus, Denmark

**Keywords:** Lived experience of losing a leg, Reflective Lifeworld Research, phenomenology, amputation, mobility, existential dimensions, caring, rehabilitation

## Abstract

This study explores the lived experience of losing a leg as described by the patients themselves post-discharge. Studies have documented that regardless of aetiology patients are faced with severe physical as well as psychosocial challenges post-amputation. However, only few studies explore in-depth the patients’ perspective on the various challenges following the loss of a leg. The study uses the phenomenological approach of Reflective Lifeworld Research (RLR). Data were collected from 24 in-depth interviews with 12 Danish patients. Data analysis was performed according to the guidelines given in RLR. The essential meaning of losing a leg is a radical and existential upheaval, which restricts patients’ lifestyle and irretrievably alters their lifeworld. Life after the operation is associated with despair, and a painful sense of loss, but also with the hope of regaining personal independence. The consequences of losing a leg gradually materialize as the patients realize how the loss of mobility limits their freedom. Patients experience the professional help as primarily directed towards physical care and rehabilitation. The findings show that the loss of a leg and, subsequently, the restricted mobility carry with them an existential dimension which refers to limitation of action space and loss of freedom experienced as an exclusion from life. Our findings demonstrate a need for complementary care and stress the importance of an increased awareness of the psychosocial and existential consequences of losing a limb.

Few studies have investigated the experience of living with the loss of a leg and how this loss influences daily life in the initial post-discharge period. To meet the challenges faced by this type of patients necessitates a deeper understanding of having to live with the consequences of lower limb amputations (LEA). Patients who have undergone an LEA represent a multifaceted group with different types of problems. The amputation is often related to chronic illness such as diabetes, vascular disease, and renal insufficiency and high rates of post-operative complications and mortality are reported (Belmont et al., 2011; Kristensen, Holm, Kirketerp-Møller, Krasheninnikoff, & Gebuhr, 2012). Furthermore, the amputation is often preceded by a long period of pain (Watson-Miller, 2006), and phantom limb pain is described after the amputation (Björkman, Arnér, Lund, & Hydén, 2010; Vase et al., 2011). Regardless of aetiology, however, the loss of a leg presents major challenges for the person concerned.

Studies have documented that the patients are faced with severe psychosocial challenges post-amputation (Donovan-Hall, Yardley, & Watts, 2002; Horgan & MacLachlan, 2004). These challenges relate to many different aspects which are associated with the loss of a leg; several surveys demonstrate that the patients suffer from anxiety, depression, restricted mobility, and social isolation (Donovan-Hall et al., 2002; Horgan & MacLachlan, 2004; Rybarczyk et al., 1992). Health-related quality of life is reported lower among amputees than controls in the first years after the amputation (Davidson, Khor, & Jones, 2010; Remes et al., 2010). Depression is also reported as a frequent response to amputation. Phelps, Williams, Raichle, Turner, and Ehde (2008) found that significant depressive and post-traumatic disorders were reported by 15%–25% of amputees and Remes et al. (2010) point out that depression symptoms were present even 3 years after amputation.

A number of studies have been carried out in order to understand the influence of lower extremity amputation on patients as seen from a psychological perspective. These studies focus on theoretical concepts such as changes in the amputees’ identity (Grobler, 2008; Senra, Oliveira, Leal, & Vieira, 2012), coping strategies (Couture, Desrosiers, & Caron, 2012; Oaksford, Frude, & Cuddihy, 2005), and the amputees’ psychological reaction to the artificial limb (Donovan-Hall et al., 2002; Murray, 2004, 2009). These studies report dominant themes such as low self-esteem and changes in self, and a struggle to accept a new identity as disabled (Donovan-Hall et al., 2002; Grobler, 2008; Murray, 2009; Senra et al., 2012).

Only a few studies specifically explore in-depth the patients’ perspective on the severe challenges following the loss of a leg. Within nursing research a grounded theory study focused on patients who have undergone a diabetes-related foot or leg amputation, five amputees described their experiences (Livingstone, Mortel, & Taylor, 2011). The authors found that despite the participants’ sense of grief, loss and shock post-operatively, they moved forward; they developed their own sense of hope and revealed remarkable ability to endure and exert control over life (Livingstone et al., 2011). This may indicate that this group of patients had dealt with their situation. Another study focusing on amputees in Taiwan described patients’ lived experience of LEA from the pre-amputation phase to 6 months after surgery (Liu, Williams, Liu, & Chien, 2010). The authors found that the amputees suffered in varying degrees and that their suffering was relatively independent of their physical pain; instead patients reported that “their suffering was endless, because they would never again be treated as a ‘normal’ person” (p. 2158).

The literature review shows that, regardless of aetiology, LEA has a significant influence on the physical and psychosocial functions of these patients. The majority of the studies are primarily cross-sectional covering specific measures of adjustment (Davidson et al., 2010; Remes et al., 2010). These studies provide important insight into the significance of psychosocial variables and their influence on the adjustment process. Also, studies within psychology have contributed to a wider insight into the complex psychological challenges facing amputees. However, studies are lacking on the influence of the loss of a leg on patients’ individual lifeworld. Consequently, there is a need for healthcare providers to explore how patients experience the severe challenges following the loss of a leg, especially in the post-discharge period in which nursing professionals are the primary caregivers. The aim of this study is to explore the lived experience of losing a leg as described by the patients themselves after discharge.

## Methodological approach

The study is carried out within the approach of Reflective Lifeworld Research (RLR). The endeavour of RLR is to describe the lived world in a way that increases our understanding of human beings through human experience (Dahlberg, Dahlberg, & Nyström, 2008). RLR is grounded in the continental philosophical lifeworld theory developed within phenomenology and hermeneutics in the first half of the 20th century by philosophers such as Husserl, Heidegger and Merleau-Ponty. The lifeworld is an intuited and common world where we act without reflection, “It is pregiven to us all quite naturally” (Husserl, 1984, p. 122). Hence, the lifeworld is tacit, implicit, and filled with meaning.

The aim of RLR is to reflect on lifeworld meanings and make the unseen or tacit aspects of human existence explicit. In this search for meanings, the researcher's openness is important. The process of bridling can accomplish this openness. To bridle means to problematize and change the “natural attitude” in favour of a phenomenological one (Dahlberg et al., 2008, p. 125), and further to focus upon and keep in check the whole process of understanding of which pre-understanding is a part (p. 130). This includes a reflective process of going beyond taken-for-granted understandings and to slow down the process of understanding by retaining an open mind in order to see the world differently and possibly be surprised by new meanings as they arise.

In line with the chosen lifeworld perspective, the term “amputee” may be seen as reductive and is therefore replaced with patients or participants in the following.

### Participants

The participants were selected from a Danish university hospital. As sampling within a phenomenological approach is concerned with gathering depth and richness of the experience, the sampling strategy is about quality rather than demographic variation and size (Giorgi & Giorgi, 2003; Norlyk & Harder, 2010). Inclusion criteria were patients who had undergone a non-malign leg amputation, were Danish native speakers, and they were discharged to their private homes. Twelve patients, eight men, and four women, aged 33–87 years participated in the study. The patients had undergone amputation of different aetiologies. Eight patients had undergone femur amputation, four a below-knee amputation and nine of the patients were referred to prosthetic training. Three of the patients lived alone while the others lived with a spouse. Five of the patients were working before the operation and seven were retired.

The patients were invited to participate in the study by their physician or nurse while they were on the hospital ward. The first author who provided written and verbal information about the purpose of the study then contacted patients who wanted to participate.

### Data collection

To obtain expressions of the patients’ lived experience, data were collected by two in-depth interviews with each patient, conducted by the first author in 2012. The interviews took place in the patients’ home. The first interview was conducted 2–4 weeks post-discharge; the second interview took place 3–4 months later. Accordingly, the post-discharge period covers the patients’ 4–5 months post-discharge. The interview process followed the recommendations of meanings-directed interviews in which the interviewer seeks to facilitate the interviewee's description of the lived experience so that the description becomes as detailed as possible and with a minimum of abstractions (Dahlberg et al., 2008). Accordingly, the interviewer approached the participants with an open mind taking nothing for granted. In the first interview, the opening question was “Could you tell me about your experiences at home after being discharged?”. Then the patients were encouraged to describe and elaborate on concrete situations from their everyday life, such as “How do you experience living with one leg?”, “What does it mean to you in your everyday life?” and “Please tell me more about how the amputation has influenced your life”. Additional questions were asked to deepen the description such as “What happened next?” or “Can you give another example of this?”. In the second interview, the patients were encouraged to elaborate or clarify some of the descriptions and comments from their first interview. All interviews were audio recorded and transcribed verbatim.

### Ethical considerations

In accordance with the basic principles for research given in the Helsinki Declaration and the Northern Nurses’ Federation (2003), the patients received written and verbal information about the purpose of the study, the right to withdraw, and the confidentiality of the data given. According to the regional ethical committee, formal approval of the study was not required because of its non-biomedical character.

### Data analysis

Interviews were analysed according to the guidelines given in RLR. The overall principle behind this phenomenological analysis is to understand each part of the text in terms of the whole text and to understand the whole in terms of its parts (Dahlberg et al., 2008). This constant movement is directed towards finding meaning; in this case, the meaning of patients’ lived experience of the existential challenges related to the loss of a leg. The analysis process focused on discovering patterns of meanings, variations, and finally an essential structure. The structure is understood as a description of the essential meaning, that is, the characteristics of the phenomenon without which it would not be that phenomenon, and its constituents. The constituents are interrelating aspects of the structure and they show the variations of the data (Dahlberg et al., 2008).

The analysis began with repeated readings of each transcript to get an overall sense of what the transcript indicated as a whole; this understanding served as a holistic, meaningful reference and background understanding within which the various parts in the individual descriptions could be understood. Then each transcript was slowly re-read and divided into meaning units. The meaning units were carefully examined to explicate all meanings that disclosed aspects of living with the loss of a leg. An intensive dialogue with the text characterized this search for meanings, moving between the interview texts themselves, and the emerging patterns of meanings. We asked questions like “What is being said?”, “How is it said?”, “What is the meaning?”. Also, we asked critical, reflective questions such as “Is this the actual meaning or can it mean something else?”. The emerging meanings that seemed to be linked were clustered into a temporary pattern of meanings. This was followed by a process of open reflection, which aimed at synthesizing the clustered meaning units into a new whole that mirrored their interrelationships and clarified the essential meaning of the loss of a leg.

First, we present the essential meaning and then its constituents. Quotes from the interviews are provided as examples of explicated meanings.

## Findings

The essential meaning of losing a leg is a radical and existential upheaval, which restricts patients’ lifestyle and changes their lifeworld dramatically post-discharge. Due to the limitations imposed by reduced physical mobility, losing a leg means loss of freedom and independence at many levels and has significant psychosocial consequences. Patients were not able to resume their former activities and contacts, which influenced their jobs, hobbies, social activities, and so on. In this new situation, patients are forced to depend on others. In brief, their lifeworld shrinks. This restriction of lifeworld is related to a sense of great losses; but is also accompanied by the hope of regaining lost territory and personal independence.

The patients find themselves in an existential limbo and strive to regain a foothold in life; they continually push themselves in an attempt to re-establish their former life and to come to terms with their new identity as physically impaired.

The disability leads to a change of role and self-image. The restricted physical mobility prevents patients from carrying out their customary roles in everyday life and leads to a more passive, recipient role. This new role is perceived as less meaningful and the patients strive to maintain their sense of personal dignity and symmetry in social relations. Mastering new ways of handling everyday routines is an important step on the road towards re-establishing a new and acceptable role in life.

Furthermore the loss of a leg involves changes in patients’ bodily awareness. Patients need to familiarize themselves with their new body and to learn new skills related to home appliances that help compensate for the lost limb.

Post-discharge, the loss of a leg involves a need for care at different levels, as care must be directed at physical care and rehabilitation as well as the difficult process of managing the transformed life.

The essential meaning is further elaborated in the following six constituents described below: Home as a confined space; Maintenance of symmetry and dignity in social relations; Reconstitution of bodily confidence; Hope and willpower as driving forces; Establishment of a meaningful role in life; Dependence on care and rehabilitation initiatives.

### Home as a confined space

Coming home was an ambiguous experience of both happiness and problems. The challenges of everyday life materialized quickly as a result of the restricted mobility. Home had suddenly changed and had become an obstacle race in which doorsteps were now huge barriers and stairs turned into mountains that had to be climbed.

Coming home, oh yes … It was like, well, great to be home; lovely … everything was as it used to be …. Great, but then I looked up at the stairs … I looked and I thought, ‘How the hell am I going to get up them? … I dreaded them. (patient 8)

The layout of the house and home appliances were absolutely crucial for patients’ experience of everyday life. Had adjustments not been made before the homecoming, the situation could become unmanageable.

I could get around in the whole house, if only I could get over the door steps. I don't understand why they haven't yet removed the doorsteps (cries). (patient 11)

It soon became routine to get about in the home in a wheelchair, although this was a huge transition. Everything was more difficult and took more time. Household chores became a burden, even down to the tiniest detail. The favourite chair might be impossible to sit in and fetching your child a glass of water or picking up something from the floor became laborious tasks.

Life has been turned upside down. Everything becomes just so difficult, nothing is easy … it isn't. When I tuck in my small son, that's upstairs, and he wants a glass of water for instance … I can't fetch it for him …. That's the way it is … I can't do it. I can't walk on crutches with a glass of water in my hand … and I can't just go set the table …. You have to have that bloody wheelchair to get around the house. (patient 3)

Physical mobility was strongly linked to freedom and independence. Having difficulties getting outside of the home contributed to the patients experiencing home as a prison, in which they felt isolated and confined.

I can easily watch the telly and read and move about the house doing bits and pieces …. But, but you are confined to these four walls. (patient 2)

### Maintenance of symmetry and dignity in social relations

Both patients and their network took on new and unaccustomed roles, which redefined patients as recipient and passive. To be unable to do the same things as before or to be a potential burden to others was associated with feelings of inferiority. Their self-esteem was threatened and they struggled to maintain symmetry and dignity in the relations they engaged in.

We went to a new shopping centre and drove past a cheese shop. ‘Hey’, I said, ‘they have some cheap cheese; let's see if they have cheese with caraway seeds’. ‘Yes’, my helper says and left me standing there while she went back to look for cheese with caraway seeds. But I wanted to see for myself if they had the cheese I like. (patient 12)

The patients experienced this as being spectators and potentially marginalized to life. Their integrity was under pressure and they tried hard to avoid being marginalized. I am who I am …. Even with a leg missing. (patient 1)

The altered appearance could cause patients to feel invisible. It was painful for patients to be ignored and patients could experience a loss of dignity. One way of maintaining dignity was to demonstrate that a one-legged person could also be active and independent.

I was buying some toothpaste which was placed fairly high up, so I just got up [from my wheel chair] and people were staring …. And the credit card terminal, it was placed so high that I couldn't reach it. So I just got up, and I kept my balance. I could sense people thinking …. ‘Gosh … can a handicapped person really do more than just sit in a wheelchair’? (patient 8)

### Reconstitution of bodily confidence

The stump was not just a shorter leg that was functional; the former, familiar body had completely changed. Normal actions such as changing position in bed were suddenly different or even difficult and could disturb a good night's sleep. The lack of bodily confidence also led to a deteriorating sense of balance.

Before I lost my leg, I could easily stand on one leg. But now … I can't even stand, I can't keep my balance on one leg. (patient 9)

The difficulty in keeping their balance could frighten and dishearten the patients whereas feeling safe and secure stimulated their courage to try out new appliances. The road to achieving confidence with new appliances involved the mastering of former bodily skills.

I still find it difficult to stand on one leg, and I am frightened every time I get into bed. They want me to use a Zimmer frame, and I cannot use it, because it is too high … and I have to hop on this leg to use the Zimmer frame. (patient 6)

The altered body led patients to experience phantom pain sensations. Through the phantom pain, the patients discovered a form of contact with the amputated leg. It was important for patients not to let the phantom pain take over but learn to accept it as part of their changed life. Familiarity with phantom pain of an acceptable level made it possible for patients to come to terms with the altered body.

### Hope and willpower as driving forces

Despite the radical changes that were life-restricting in varying degrees, the patients also experienced that the altered conditions of life were connected with hope, for example, the amputation could be the only way out of deadlock and a hopeless situation

I haven't been able to walk properly for two years now because of pain. In the end I didn't have a life. I was in constant pain and finally I was afraid of going to bed because I would wake up in a few minutes in a lot of pain. It was a nightmare at the end … something had to be done. (patient 12)

Although the patients were anxious about the future, they tried to keep a positive attitude towards life. They struggled to find the willpower and stamina to carry on with life, and they worked with determination towards increasing their mobility and freedom of action. The road to independence involved continually exploring and expanding personal boundaries. By fulfilling their goals, the patients produced the willpower to constantly challenge themselves in order to regain the maximum of their former territory and mobility. The alternative was resignation which implied giving up and accepting marginalization and restricted action space.

It is my goal to get back to work again…. It may not be realistic, but you need to have a goal … if you don't have goals, you might as well give up. (patient 3)

Prosthesis patients experienced that the prosthesis gave them hope and it became a lifeline to their former active life. The prosthesis symbolized their hope of walking again, of becoming more independent, and returning to their former life.

I believe that when I get my prosthesis, I will be able to walk again and that is my goal. I won't be able to do everything myself again, I know that, but certainly as much as possible. (patient 5)

Complications, for example, delayed wound healing, postponed the rehabilitation process and put a hold on the patients’ ambitions of a speedy return to everyday life. The delayed rehabilitation process was experienced as being in a vacuum in which time stood still and life was on standby. This situation was difficult to cope with and had a negative influence on patients’ ability to maintain hope and willpower.

### Establishment of a meaningful role in life

For patients, establishing a new role and becoming whole again involved reconciliation with the altered life conditions. This process was time consuming and could not be forced as it involved accepting that, although life had changed forever, it was still worth living.

Before, when I had my rollator, I could go to the shopping centre or anywhere I wanted, you know, but well … that is all over now, and it can't be helped …. So now I am trying to pick myself up again. But I'll never be whole again … one doesn't. Never … but I have learned to live with it. (patient 6)

The patients fought hard to re-establish themselves at many levels in life, from reviving previous interests to trying to maintain personal relationships. Re-establishing oneself in life required great willpower and creativity; overcoming even the tiniest obstacle was an important victory.

We have a caravan, it is our refuge and when I could manage to even think about walking around on crutches, we went camping. It was a little difficult at first, getting into the caravan with a pair of crutches, but one of our friends made a small step for me, so I succeeded, and that was the most important thing. (patient 4)

The hope of re-conquering former interests, habits, goals, and dreams was a way to establish a feeling of normality and a certain degree of freedom. However, to regain the former life involved being confronted with new and limited possibilities, which was painful.

I was down at the golf course driving a buggy behind some of the people I normally play with …. I almost envied them their legs … I sat there looking at their legs … I was looking at their bloody legs; I didn't even notice their golfing … I was so envious of their legs. No, it is bloody hard to accept. (patient 9)

### Dependence on care and rehabilitation initiatives

The help offered for restitution either increased or decreased the patients’ quality of life. The right type of help could increase patients’ quality of life a lot. A handicap vehicle, such as a car or a crosser, restored a certain degree of freedom and action space. In reverse, not having the right appliances reduced the potential for independence, as patients would have to rely on the help of others. This meant they could feel restricted and cut off from the outside world. One patient describes his experience as follows:

My wings are clipped; I can't really go anywhere … just around the block. I grew up in this neighbourhood and I'd very much like to get around by myself. (patient 1)

Physiotherapy was a very important rehabilitation initiative that gave patients hope for the future and provided a form of structure in uncertain circumstances. Training was a symbol of not having given up on life. To remain motivated, it was important that patients experienced the training as meaningful.

They all say 'come on, now, pick up your crutches and walk a bit. You've got to start using your crutches. Well, where can I go? Go into the kitchen, and do what exactly? I can't do anything, when I use my crutches, can I? I've got my hands full and it feels like they [nursing professionals] are asking me to do a lot of things I can't do anyway. (patient 4)

Patients found it hard to come to terms with the existential challenge of losing a leg and having to live with reduced mobility; they also experienced that professional help and support primarily addressed the mere physical aspects of their new situation.

I sometimes wonder … no … well. They [professionals] might try to think a bit more about what it is like to be me. (patient 10)

In other words, although the patients got practical help from healthcare providers they felt alone and left to themselves in circumstances that were extremely difficult to deal with.

## Discussion

This study illustrates how the loss of mobility disrupts the patients’ lifeworld in several ways. Patients’ relationship to the world was disturbed and this was experienced as a sense of exclusion from life. In spite of a general feeling of being all right, the reduced action space and limited freedom were experienced as existential losses. Consistent with other studies (Donovan-Hall et al., 2002; Grobler, 2008; Horgan & MacLachlan, 2004; Senra et al., 2012), this study reveals psychosocial and physical challenges related to the patients’ adjustment process including change of self-image, social isolation, despair, and a struggle to come to terms with a new identity as physically impaired. Our findings provide important additional insight related to the above studies through the participants’ elaboration on the existential dimension. This study shows that the physical loss of a leg and the subsequent restricted mobility carry with them an experience of loss of action space and freedom. Furthermore, the study reveals how the sense of loss escalates as patients experienced loss in many forms. These findings may explain why Remes et al. (2010) and Davidson et al. (2010) found that low quality of life primarily is related to restricted mobility.

This study also highlights that mobility has several meanings: first, mobility involves the physical dimension of moving from A to B, which affects action space; second, mobility involves an existential dimension that influences patients’ social and personal space. This existential dimension of mobility can be further understood by drawing on the philosophical work of Merleau-Ponty (2005). In an account of people living with physical impairment, Merleau-Ponty illustrates how loss of ability interrupts everyday life because the relationship between body and world is disturbed (Merleau-Ponty, 2005). As expressed by Merleau-Ponty (2005), “The body is the vehicle of being in the world” (p. 94). Thus, we do not *have* a body, we *are* our body and consequently the body connects us to the world. Through the body, the surrounding world becomes meaningful. As stressed by Merleau-Ponty (2005), the body carries out all living actions of which many are without reflection as they are embodied knowledge.

Toombs (1993), herself a phenomenologist living with physical impairment, further explains that bodily dysfunction causes a disturbance between embodied knowledge and world because disability is the “inability to engage the world in habitual ways” (p. 62). This study shows that the patients now viewed the world through their reduced body and had lost their former ability to live the body unreflectively. The body became an object that prevented them from living their former life, and the patients experienced a kind of alienation from their body. According to Toombs (1993), illness disturbs the relationship between body and self. She explains that there is no perceived separation between body and self at the level of the lived body. A dysfunctional body, however, is perceived as a defective “physico-biological thing” (p. 71) meaning that dysfunction necessarily incorporates not only a threat to the body but also a threat to one's very self (Toombs, 1993). An increased awareness of this threat to patients’ self can help healthcare professionals understand the vulnerability of the patients and draw attention to the ways in which they are affected by the disruption between body and self.

As shown in our findings, the loss of a leg can be understood as bodily dissonance characterized by a conflict of wanting and not being able to, expressed by Dahlberg et al. (2008) as a sense of “lacking bodily autonomy” (p. 45). We found that this lack of bodily autonomy meant that the patients’ surrounding world looked and felt different. Accordingly, our findings add to the understanding of the importance of bodily autonomy by showing that lack of bodily autonomy not only meant a limitation of action space but also a loss of freedom and disruption of the patients’ social world. Everyday actions, which were formerly taken-for-granted, now presented themselves as problems related to the reduced body. The patients desired to re-establish their former lifestyle and hoped to regain personal independence.

The findings also show that accessing the world with a lost limb is a struggle which requires that patients constantly challenge and continually push themselves in order not to give up on life. In our study, the patients refused to be marginalized and to take on the passive role of a victim. The patients, in other words, had resources. However, the professionals did not always support these resources. The patients perceived the professional help as practical and primarily directed towards physical care or rehabilitation while a holistic approach to the consequences of losing one's mobility was not included. The findings show that the one dimensional and instrumental approach leaves the patients feeling alone in the difficult process of managing their transformed life.

Patients’ experience of professional help as predominantly practical is further supported by Liu et al. (2010). The authors found that patients perceived their care providers as focused on the surgery, avoidance of complications, and wound healing during the early post-operative phase. This clashed with the patients’ focus on coping with fear and anxiety, questions about the future, the impact of the amputation and the “nuts and bolts” of what they should do and expect after amputation (Liu et al. 2010).

The results of the present study highlight the necessity of meeting patients who experience the loss of a leg with a more holistic approach. Meeting the challenges faced by this group of patients necessitates a deeper understanding of the consequences of having to live with the loss of a leg. Recently, the concept “lifeworld-led care” has been developed in order to enable healthcare professionals to attend to lifeworld directed ways for caring (Dahlberg, Todres, & Galvin, 2009; Todres, Galvin, & Dahlberg, 2007). Lifeworld-led care rests on a philosophical informed framework to care that acknowledges the complexities of personhood, health, and illness. Embedded in this approach is an existential view of well-being that focuses on what makes well-being an experiential possibility and includes the dimensions of freedom and vulnerability (Dahlberg et al., 2009). Hence, lifeworld-led care is not only holistic; it is also directed towards patients’ existential well-being. This approach to care seems to be particularly relevant as our study points to the need for individualized support concerning the existential challenges faced by the patients who find themselves trapped and isolated in the difficult process of managing their altered life.

## Conclusion and implications for practice

This study illustrates how the lifeworld of patients is reduced as a consequence of the loss of mobility. Life after discharge is associated with a feeling of despair and a painful sense of loss, but also with a hope of regaining personal independence. The consequences of losing a leg gradually materializes as the patients realize how the loss of mobility limits their freedom.

The findings show that the loss of a leg and, subsequently, the restricted mobility carry with them an existential dimension due to limitation of action space and loss of freedom which is experienced as an exclusion from life. Patients experience the professional help as primarily directed towards the physical and practical aspects of their situation. Our study shows that this limited approach is of concern and that patients require complementary care.

In particular, healthcare providers and nurses must facilitate patients’ transition towards life as physically impaired. Implications for healthcare practice need to include an increased awareness of the psychosocial and existential consequences of losing a limb. The recently developed concept of lifeworld-led care may help healthcare providers to recognize the various forms of existential challenges faced by patients in this situation. However, there is a need for the development of research-based rehabilitation programmes that provide health professionals with strategies to support these patients in their efforts to cope with their transformed life.
